# Influence of pre-slaughter fasting time on weight loss, meat quality and carcass contamination in broilers

**DOI:** 10.5713/ajas.20.0560

**Published:** 2020-11-03

**Authors:** Ge Xue, Silu Cheng, Jingwen Yin, Runxiang Zhang, Yingying Su, Xiang Li, Jianhong Li, Jun Bao

**Affiliations:** 1 College of Life Science and Technology, Northeast Agricultural University, Harbin, Heilongjiang 150030, China; 2 College of Animal Science and Technology, Northeast Agricultural University, Harbin, Heilongjiang 150030, China

**Keywords:** Arbor Acres (AA) Broiler, Pre-slaughter Fasting, Weight Loss, Carcass Contamination, Meat Quality

## Abstract

**Objective:**

An experiment was conducted to determine the appropriate fasting time prior to slaughter for broilers in floor-feed and scatter-feed mode.

**Methods:**

On 21 d since hatching, 120 Arbor Acres broilers were divided into floor-feed and scatter-feed groups, chicks from each group were further assigned to feed withdrawal treatments for 0, 4, 6, 8, and 10 h. Some resultant indicators such as carcass contamination, body weight loss, meat quality of 54-day-old broilers were measured.

**Results:**

It appears that longer feed withdrawal increased weight loss, lightness, drop loss of meat but reduced pH. A significant higher weight loss and lightness for both floor-feed and scatter-feed chicks coincided after 6 to 10 h feed withdrawal (p<0.05). pH for breast muscle at 45 min postmortem reduced when chicks of scatter-feed were fasted 6 and 10 h, while the reduction of floor-feed group occurred only in 10 h (p<0.05). A noticeable effect of feed withdrawal on drop loss occurred after 10 h fasting in scatter-feed of which drop loss were significantly higher than that for other groups including control (p<0.05). The change of contamination propensity revealed that 6 to 10 h fasting significantly reduced the likelihood of carcass contamination under both floor-feed and scatter-feed (p<0.05). Net weights of intestinal contents for gizzard were significantly reduced after feed deprived for 10 h in floor-feed and 6 and 10 h in scatter-feed (p<0.05). The decrease for whole intestine occurred after floor-feed broilers have been without feed for more than 4 h, scatter-feed broilers for more than 8 h (p<0.05).

**Conclusion:**

On the premise that poultry product properties and welfare were not significantly damaged, proper fasting time could reduce carcass contamination. Current data implied that 6 h fasting was recommendable for both floor and scatter feed pre-slaughter broilers.

## INTRODUCTION

The day of slaughter, poultry are exposed to many handlings processes including feed withdrawal, catch, lairage, transport and shackle, which both individually and (or) additively can contribute to animal losses and substandard animal welfare at slaughter [1]. Feed withdrawal before catch and live haul is a recommended practice for on-farm preparation of poultry and livestock in order to reduce carcasses pollution and ensure meat hygiene due to lower risk of manure excretion during transportation and gut contents spillage during carcass evisceration [2,3].

Generally, 10 to 11 h removal of feed and water from market-aged broilers is performed on farms and abattoir including 5 h fasing before lairage at least, 2 to 3 h capture, 2 h transport and 1 h rest. The actual time fasting may be longer during operation [4]. Short-term fasting or non-fasting before slaughter are associated with carcass contamination, increased transport stress and feed waste, resulting in increased processing costs and impaired economic benefits [2,5,6]. Simultaneously, long-term fasting as a pre-mortem stressor is also a carcass and meat quality issue as it may cause carcass depreciation result from weight losses, and meat quality defects due to abnormal post-mortem muscle acidification [7]. Nijdam et al [8] documented weight loss of poultry after fasting and transportation was 0.42%/h, about more 0.3%/h than that of poultry without fasting before transportation. Brizio et al [2] and Carneiro et al [9] demonstrated the length of fasting affects initial glycogen loading at the time of postmortem anaerobic glycolysis, and glycogen content in muscle tissue determines the rate and extent of pH decline during the onset of rigor mortis, further affects attractive characteristics of meat. Besides, in the study of Caffrey et al [6], the mortality rate of broilers during transportation increased whatever fasting time before crating is too long or too short and the lowest risk resulted from fasting for 6 to 9 h.

From above, it is sufficiently essential to ascertain optimum time of fasting in order to maintain acceptable welfare conditions for chicks, ensure optimal, consistent, uniform carcass and meat quality and maximize the economic benefits. In addition, although a number of studies have been reported on feed withdrawal, these usually focus on fasting methods and influence of early fasting [9,10], more researches are needed in optimum fasting time. The objective of the present study was to determine the optimal fasting time for floor-feed and scatter-feed broilers by testing the effects of different fasting time preslaughter on weight loss, meat quality, carcass contamination in broilers.

## MATERIALS AND METHODS

### Experimenal animals and design

All experiments and procedures described in this study comply with the Guidelines of Northeast Agricultural University Rules concerning Animal Care and Use and have been approved by the Northeast Agricultural University Animal Care and Use Committee (IACUCNEAU20150616). A total of 150 healthy one-day-old Arbor Acres male broilers with 39.59 g average initial weight (standard error = 2.12) were brood on the indoor floor, established with a 10 cm thick sawdust bedding, a moisture proof film, with a feeding density of 8 to 10 birds/m^2^. Brooding was conducted in a chicken house (relative humidity, 60% to 70%) using natural ventilation (ventilation windows). Artificial temperature control (electrothermal film heating, 34°C to 35°C the first week, reduced by 2°C to 3°C every week) and artificial light (incandescent lamps, 23 h the first 3 days, 7:00 am to 5:00 pm from the 4th day) were used. Immunization and disinfection were performed according to routine procedures.

A total of 120 healthy three-week-old broilers with a mean body weight of 617.76 g (standard error = 7.79) were selected and randomly divided into two groups (floor-feed and scatter-feed groups). Floor-feed broilers (n = 60) continue to be raised on the indoor floor with a feeding density of 10 birds/m^2^ but were exposed to natural light, artificial and natural ventilation (electric fans are provided), stable temperature (22°C to 23°C daytime, 16°C to 18°C night) and humidity (48% to 50%). Scatter-feed broilers (n = 60) had outdoor access from a plastical shed (a metal frame of 2 m long, 1.5 m wide, 1.2 m high, 20 cm above the ground and 5 cm padding established) to an activity space (a rest area with sunshade net provided) during daylight hours (from 7.00 am to 5.00 pm) and were forced into the shed at night. The birds were exposed to natural light, stable temperature (22°C to 26°C daytime, 14°C to 17°C nighttime) and humidity (30% to 40%). And the stocking density was 10 birds/m^2^.

In both feeding methods, bedding were covered with a thin new layer every 4 to 5 days. All birds had free access to and water during the rearing period. Immunization and disinfection are performed according to routine procedures. Commercial broiler diets were fed to the birds, including starter diet (metabolizable energy of 12.1 MJ/kg and crude protein of 21.0% for 1 to 3 week of age) and finisher diet (metabolizable energy of 12.6 MJ/kg and crude protein of 19.0% for 4 to 6 week of age).

At 53 day, 50 healthy broilers with similar weight of 2,243 g (standard error = 46.65) from floor-feed and 2,176 g (standard error = 56.36) from scatter-feed were weighed and separated randomly into 5 treatment groups (2 replicates, 5 birds each), respectively. Feed tanks and gutters removed at 20:00, 22:00, 24:00, 02:00, 06:00 to meet, respectively, 10, 8, 6, 4, and 0 h fasting periods. Artificial light was provided for 10 h (from 20:00 pm to 06:00 am the next day). At 06:00 h on d 54, 3 chickens were randomly selected from each replicate, marked with a livestock paint, placed in crates (density of 5/box) and transported to the laboratory. Birds were weighed and killed by cervical dislocatio after 3 h transport on the road, 1 h lariage in laboratory and 12 s shackling on hook. Contaminate propensity was defined after skinning before dissection. Breast muscles were taken for subsequent meat quality test, and proventriculus, gizzard and whole intestine were taken for observation of intestinal cleansing.

### Sample collection and index determination

#### Weight loss

Weight loss (Ws) was calculated from weight difference between before fasting (W_1_) slaughter (W_2_), according to the following formula: Ws = W_1_−W_2_.

#### Meat quality

pH for breast muscle at 45 min (pH_1_) and 24 h (pH_2_) postmortem was measured in the breast muscle (pectoralis major) using a pH meter (Delta-320, Mettler Toled, Shanghai, China) provided with a specialized probe and temperature control system. This apparatus was calibrated with three standard fluids solution: pH = 4.003, 6.864, and 9.182 following a procedure provided by manufacturer. Between the measurements the muscle was stored at 4°C wrapped with a plastic wrap.

Lightness (L*), redness (a*), and yellowness (b*) value were determined at 1 h post mortem using a Chroma meter (CR-400, Konica Minolta, Osaka, Japan) on the CIE Lab system. The left pectoralis minor muscle was cut with a thickness of no less than 1.5 cm. The tip of the chromameter measuring head was placed flat against the medial surface, and the colorimeter was calibrated using the specific white board before measurement. Each value was an average of measurements from 3 different sits of the sample.

The breast fillets (5 cm×3 cm×2 cm) is weighed after slaugh ter (W_1_), hung on a 50 mL cold storage tube at 4°C for 24 h, then reweighed (W_2_) after wiped off their surface liquid. Drip loss was calculated as a percentage of weight loss during storage as follows: Drip loss (%) = (W_1_−W_2_)/W_1_×100%.

Warner-Bratzler shear force analysis was performed im mediately after drip loss measurement. Meat samples was placed in vacuum-package bags, cooked in water bath (80°C) until the core temperature reached 75°C and remain for 10 min, then trimmed into a cylinder (2.5 cm long, 10 cm diameter). Shear force was measured on a tenderometer (C-LM3, College of Engineering, Northeast Agricultural University, Harbin, China). The Warner-Bratzler single blade sheared the meat column in the direction perpendicular to the muscle fiber, with the shear velocity of 5 mm/s. The maximum shear force was record, averaging 5 readings for each sample.

#### Carcass pollution

After peeling and plucking and before eviscerating, the propensity to contaminate was judged and record subjectively according to research of Zuidhof et al [11]. The position of poultry abdomen (3 cm from the anus) was continuously pressed 3 times by an experimenter using uniform force. Fecal spillage was observed, assessed by two experimenters on a scale from 0 to 5, with category 0 being no fecal discharge and category 5 being the maximum discharge (Table 1). Contaminate propensity was the average of these observations.

After the broilers was cut open, proventriculus, gizzard and whole intestine (excluding the rectum) were taken out, weighted immediately full and empty. Weight of contents was calculated by difference before and after contents were squeezed out.

### Statistical analysis

SAS (ver. 9.1, SAS Institute, Cary, NC, USA) was used for statistical analysis. All data were tested for normal distribution using the Univariate analysis before statistical analysis. Least square mean and standard error (mean±standard error of the mean) of each treatment for weight loss, muscle pH, meat color, drop loss, shear force, contamination propensity, weight of gastrointestinal content were calculated by applying two-factor analysis of variance (ANOVA). And intergroup and intra-group differences between the same and different feed manners were evaluated by Duncan’s multiple comparison at a significance threshold of 5%.

The ANOVA model was as follows:

$${\text{Y}}_{\text{ijk}}=\mu +{\text{E}}_{\text{i}}+{\text{W}}_{\text{j}}+{\left(\text{EW}\right)}_{\text{ij}}+{ɛ}_{\text{ij}}.$$

Y_ijk_, individual observed value; μ, mean of population; E_i_, pattern effect; W_j_, length effect; (EW)_ij_, interaction of pattern and length; ɛ_ij_, random error.

## RESULTS

### Weight loss

Table 2 shows the average results for body weight loss of broilers evaluated after 0, 4, 6, 8, and 10 h fasting under different rearing forms. Compared with 0 and 4 h groups, body weight loss was obviously higher in 6, 8, and 10 h groups with the hightest loss seen in 10 h group of the two feeding manners (p<0.05). There was higher weight loss in 10 h group (p<0.05) and lower weight loss in 6 h group (p<0.05) compared with 8 h fasting group of floor-feed. Weight loss in 10 h group of scatter-feed was significantly higher than that in 6 h group (p<0.05). Body weight loss of floor-feed was much more in 10 h groups compared with scatter-feed (p< 0.05). While there was no significant interaction between feeding manner and fasting length on weight loss of broilers (p>0.05).

### Indexes of meat quality

Variation of meat quality indicators (pH_1_, pH_2_, L*, a*, b*, drip loss, shear force,) of broilers under different fasting time are shown in (Table 3). With the exception of pH_1_, L*, drip loss, the other meat traits did not vary significantly according to the length of the feed withdrawal (p>0.05). pH_1_ in 10 h group of floor-feed were significantly lower than that in 0, 4, and 6 h groups (p<0.05). And pH_1_ in 6 and10 h groups of scatter-feed was significantly higher than that in 0 and 4 h groups (p<0.05). Drip loss in 10 h group of scatter-feed was higher than that in 0, 4, and 6 h groups (p<0.05). Meat color L* value was clearly higher in 6, 8, and 10 h groups compared with 0 and 4 h groups of floor-feed with the highest value seen in 8 h group (p<0.05). L* value in 6, 8, and 10 h groups of scatter-feed was significantly higher with the highest value seen in 10 h group compared with non-fasting group (p<0.05). Significant difference only was observed in L* value between the two feed manners, and L* value of non-fasting group was distinctly higher in floor-feed compared with scatter-feed (p< 0.05). There is no significant interaction between feeding manner and fasting length on all these indicators of broilers (p>0.05).

### Indexes of carcass contamination

Table 4 presents the effects of feed withdrawal on indicators of carcass pollution (contamination propensity, weights of intestinal contents for proventriculus, gizzard, and whole intestine) as a function of length of the period (0, 4, 6, 8, and 10 h). Fasting time had an important effect on contamination propensity since contents of gizzard and whole intestine overall decline with increasing fasting time. Contamination propensity of the two feeding manners were significantly lower in 6 h group compared with non-fasting group (p< 0.05) and 10 h fasting could reduce contamination propensity to a new level (p<0.05). Significant lower contents of gizzard was found in l0 h fasting group of floor-feed compared with 0 and 4 h fasting groups (p<0.05). And compared with non-fasting group of scatter-feed, contents of gizzard in 6 and l0 h groups was pretty lower (p<0.05). The contents of whole intestine in 4, 6, and 8 h groups of floor-feed were lower contrast to non-fasting group (p<0.05) but higher contrast to 10 h fasting group (p<0.05). Contents of whole intestine in 8 and 10 h groups was significant lower compared with non-fasting group of scatter-feed (p<0.05). Difference in contents of gizzard and whole intestine was acquired between the two feed manners with lower gizzard contents in 0, 8, and 10 h groups (p<0.05) and lower whole intestine contents in 10 h groups of floor-feed (p<0.05). There was no significant interaction between feeding manner and fasting length on those indexes of carcass contamination (p>0.05).

## RESULTS AND DISCUSSION

### Effect of the length of preslaughter fasting on weight loss of broilers

The live weight loss was significantly increased as length of feed withdrawal was extended from 0 to 10 h prior to slaughter for both floor and scatter groups and 10 h fasting resulted in a total of 70.70 g loss (approximately 3.44%) in foor-feed group and 46.90 g (approximately 2.16%) in scatter-feed group. This synergistic relationship and rate of weight loss are in agreement with the reports by Abdullah et al [12] who found that body weight loss of broilers (Ross 308 and Hubbard) significantly increased linearly with fasting time from 1.65% at 4 h to 3.50% at 12 h, and Zuidhof et al [11] who observed that 0.22%/h to 0.56%/h weight loss of broilers was on account of fasting before slaughter and the maximum weight loss occurred after 10 h fasting. As for the reasons, previous reports have reported excretion of gastrointestinal contents, water loss in muscle tissue and nutrients consumption for oxidative metabolism are three major reasons for weight loss of animals related to fasting [13–16]. In our study, a significant live weight loss for the two feed groups coincided after 6 h feed withdrawal. And a new significant level was showed every 2 h of foor-feed broilers, every 4 h of scatter-feed broilers over 6 h fasting. Kim et al [14] verified that the weight loss depending on a short period of feed withdrawal (approximately 6 h) prior to slaughter was due to the changes in digestive tract content mostly, which means no loss of carcass yield. However, it have been reported reduction in moisture content occurs at the beginning of fasting and goes to be obvious over 6 h fasting [13]. In addition, 5 h fasting was sufficient to stimulate lipolysis and up-regulate the genes related to fatty acid oxidation [15]. Furthermore, Arsenault et al [16] has found that basically empting of gastrointestinal contents requires 8 to 12 h fasting. Therefore, we speculate that the significant live weight loss for the two feed groups coincided after 6 h feed withdrawal was mainly related with expulsion of gastrointestinal contents. While, the new significant level showed over 6 h fasting probably because of not only evacuation of gastrointestinal contents but also significant muscle dehydration, nutrients consumption, which may lead to obvious edible yield loss. To ensure the carcass yield was not affected, 6 h fasting is recommended for floor and scatter feed chicks.

### Effect of the length of preslaughter fasting on meat quality of broilers

It has been widely reported that the effects of both changes in muscle pH during the onset of rigor mortis and ultimate pH on muscle protein degeneration, myofibrillar filament spacing and muscle cell contraction are the basics of meat quality (raw or cooked) [17]. Our results showed that the increase of fasting time resulted in a downward trend in breast meat pH_1_ with a remarkable reduction observed in floor-feed chicks fasted for 10 h and floor-feed chicks fasted for 6 and 10 h, but no marked difference in pH_2_, which consistent with the study of Kotula and Wang [18] and Tougan et al [3]. We speculate declined 45 min-pH was driven from the fact that long-term food deprivation could lead to glycogen depletion, glycolysis, followling accumulation of lactic acid and phosphoric acid in muscle tissue [8,19]. As to why there is no difference in 24 h-pH, it may because when lactic acid is accumulated to a certain extent, glycogenase will gradually lose its activity lead to a stable acid environment ultimately.

Most water is stored in myofibrils, accounting for about 80% of total muscle weight [20]. Water-holding capacity, the most important quality trait, commonly is measured with drip loss. High water loss is identified as a severe meat product issue as it lead to damaged palatability and appearance of meat and high economic loss of animal husbandry [21]. Our results showed that 10 h fasting in scatter-feed caused severe drip loss, which is similar with the study of Tougan et al [3] who observed that 12 and 24 h of feed withdrawal lead to reduced water holding capacity of indigenous chicken in Benin. As discussed widely, metabolic rate of pH had a significant effect on water-holding capacity [20,22,23], we speculate that the higher drop loss in scatter-feed broilers fasted for 10 h is due to the lower pH_1_. On the one hand, a driving force for drip loss is provided due to smaller fine filament spacing and muscle fibers volume at a lower pH [20]. It was reported that muscle fibers volume was reduced by about 15% when pH dropped from 7 to 5, which was a crucial cause for the drip loss of pale, soft, and exudative meat [22]. On the other hand, the decreased pH_1_ value in our study was closer to the isoelectric point of muscle protein (5.0 to 5.5) [23], which lead to lower total static charge of protein and water-holding capacity. As can also be noticed, water-holding capacity at 24 h postmortem of chicken meat has higher correlation with the 45 min-pH than with the 24 h-pH, which is understandable as although there was no difference in pH_2_, the lower pH_1_ indicated the meat experienced a period of a more acidic environment during storage.

Muscle tenderness, a generalization of the structural pro perties of various proteins in muscle [24], serves as an important indicator for consumers to evaluate meat acceptability [20]. It is usually measured by shear force, the lower shear force, the higher tenderness. The main influencing factors for tenderness include pre-slaughter ones (genetics, rearing, management, etc.) and post-mortem ones (meat pH, meat maturation, cooling, etc.). Different from the report of Tougan et al [3] in which shear force reduced significantly, the indicator did not differ after feed removal of different time in our study. The inconsistency may be caused by diverse animal species and treatment methods, but the specific reason needs further study.

Meat color reflects biochemical, physiological and micro bial changes in muscles and is a key attribute of consumer acceptability [25]. For red muscles, the most important meat color indicator is a* value (redness), while for white muscles like chicken, that is L* value (brightness). Thus more attention should be paid to the change of L* value in our study. It has been reported that when L*>53, the meat color tends to be lighter, 48<L*<51 indicates that meat color is normal and L*<46 implies that meat color tends to be dark [26]. According to previous report, shading and uniformity of meat color are primarily determined by content, distribution and chemical state of myoglobin and hemoglobin, pigment substances in muscle [25], Moreover, it also relates to light refraction, scattering and absorption within a muscle fibre [25,27]. Our study showed that the length of fasting had no significant impact on meat color a* and b* value of broilers, which concides with the results of Pereira et al [27]. But the conspicuous increase in L* value under floor-feed or scatter-feed after fasting for over 6 h was observed. As previously reported, reducing the pH environment of a muscle fibre induces increased global brightness and more light scattering within fibre by narrow of myofilament lattice spacing, shorten of sarcomere and some irreversible change like precipitation of particles in sarcoplasmic fluid, or increase scattering from certain structures by decorating them with aggregated protein [28]. Higher light refractive index of meat is also associated with the more drop loss at low pH because because incident light could be reflected or scattered by the moisture of the meat surface [25,28]. Furthermore, heme in myoglobin and hemoglobin is the primary absorber of light at certain wavelengths. Reducing of pH leads to lower light absorption of myoglobin and whiten meat [28,29]. Therefore, we hypothesized that the whitened flesh in our study result from the lower pH_1_ and increased drop loss.

### Effect of preslaughter fasting time on carcass contamination of broilers

Food-borne diseases have a adverse impact on public health and economic benefit [30]. A variety of pathogenic microorganisms exist in faeces, digestive tract and gallbladder of poultry, many of which are zoonotic pathogens such as Salmonella and Campylobacter [31]. Thus severe cross-contamination of edible parts may occurred due to fecal spillage, digestive tract rupture or bile overflow during slaughter. It has been observed 5.5% to 25.2% of digestive tract would rupture when broiler corpus is removed [30], 2% to 34.0% of broiler carcasses were contaminated during slaughter [32]. Carcasses contamination has been regarded as a major issue according to the public health standards of many countries, and concerned by more and more animal husbandry workers because it undermines carcass production and meat quality, increases market complaints and labor demand. Therefore it is necessary to control carcass contamination rate during slaughter, while adjusting length of fasting was one of the effective measures [2]. In the study, net weights of intestinal contents for gizzard were significantly reduced after feed deprived of 10 h in floor-feed and 6 and 10 h in scatter-feed. The decrease for whole intestine occurred after floor-feed broilers have been without feed for more than 4 h, scatter-feed broilers for more than 8 h. Due to the emptying of gizzard and whole intestine, contamination propensity significantly deceased after 6 h fasting in both floor-feed and scatter-feed groups, which reduced to a new marked level after 10 h fasting due to the emptying of gizzard and whole intestine contents. According to the previous reports, broilers carcasses after 4 h fasting were severely contaminated [11]. Intestinal excretion was still relatively abundant when the length of fasting was less than 6 h, resulting in more excretion during transportation and increasing possibility of intestinal contents leakage [6]. The digestive tract was near completely clean and the intestine was still sturdy after 8 to 12 h of pre-slaughter fasting [16]. Moreover, it was also reported that too long fasting (>12 h) might cause bile reflux, intestinal and gallbladder rupture, resulting in increased pollution rate [2]. Thus, it can be seen that 6 to 10 h fasting is optimal to reduce carcass contamination and ensure meat safety which is in line with our study.

## CONCUSION

In summary, proper fasting before slaughter can reduce the risk of poultry stress and carcass pollution, alleviate the decline of meat quality and improve production efficiency. Fasting for 6–10 h largely reduce the likelihood of carcass contamination under both floor-feed and scatter-feed. Fasting for 6–10 h could upside L* value and weight loss of both floor-feed and scatter-feed broilers; 10 h fasting could seriously increase pH_1_ and drop loss of broilers in floor group. It is suggested that the best pre-slaughter fasting time for floor and scatter feed broilers is 6 h with a little impact on meat quality.

## Figures and Tables

**Table 1:** Contamination propensity scale

Grade	Volume of feces excretion (mL)
0	0
1	0–2
2	2–4
3	4–6
4	6–8
5	>8

**Table 2:** Effects of the length of fasting on weight loss of broilers

Indexes	Raising pattern	Length of fasting (h)1)					Effect2)		
	
		0	4	6	8	10	Pattern	Length	Pattern×length
Weight loss (g)	Floor-feed	12.90^d^±0.68	15.50^d^±0.34	21.60^c^±0.41	45.10^b^±2.69	70.70^a^^x^±5.28	*	*	-
	Scatter-feed	13.50^c^±1.40	15.20^c^±1.30	22.50^b^±0.75	39.10^ab^±2.61	46.90^a^^y^±2.79			

1) The data are presented as means±standard error of the mean.

2) - means p>0.05, * means p<0.05.

a–d Different superscripts in a row indicate significant difference at p<0.05.

x,y Different superscripts in a column indicate significant difference at p<0.05.

**Table 3:** Effect of the length of fasting on meat quality of broilers

Indexes	Raising pattern	Length of fasting (h)1)					Effect2)		
	
		0	4	6	8	10	Pattern	Length	Pattern×length
pH_1_	Floor-feed	6.60^a^±0.03	6.61^a^±0.06	6.55^a^±0.01	6.53^ab^±0.03	6.39^b^±0.03	-	*	-
	Scatter-feed	6.74^a^±0.04	6.88^a^±0.03	6.54^b^±0.01	6.62^ab^±0.03	6.52^b^±0.04			
pH_2_	Floor-feed	6.20±0.02	6.20±0.02	6.23±0.02	6.27±0.01	6.20±0.01	-	-	-
	Scatter-feed	6.25±0.01	6.34±0.01	5.27±0.19	6.28±0.02	6.31±0.02			
Drip loss (%)	Floor-feed	3.82±0.43	2.56±0.06	2.56±0.10	5.65±0.49	5.78±0.34	-	*	-
	Scatter-feed	3.52^b^±0.33	2.26^b^±0.13	2.53^b^±0.10	4.69^ab^±0.31	7.32^a^±0.60			
Shearing force (N)	Floor-feed	2.29±0.17	1.44±0.10	2.30±0.20	1.80±0.15	2.26±0.11	-	-	-
	Scatter-feed	1.95±0.17	1.86±0.16	2.05±0.15	2.10±0.12	1.96±0.18			
L*	Floor-feed	50.51^b^^x^±0.20	48.58^b^±0.40	54.36^a^±0.42	54.85^a^±0.39	54.20^a^±0.36	*	*	-
	Scatter-feed	46.48^b^^y^±0.15	49.18^ab^±0.51	51.68^a^±0.33	52.00^a^±0.52	52.59^a^±0.18			
a*	Floor-feed	1.26±0.06	1.15±0.15	1.38±0.14	1.47±0.18	1.34±0.12	-	-	-
	Scatter-feed	1.05±0.10	1.19±0.14	1.11±0.12	1.02±009	1.15±0.06			
b*	Floor-feed	12.61±0.43	12.99±0.40	14.01±0.40	10.88±0.41	10.39±0.24	-	-	-
	Scatter-feed	12.35±0.22	13.08±0.40	13.26±0.28	11.21±0.44	12.47±0.34			

1) The data are presented as means±standard error of the mean.

2) - means p>0.05, * means p<0.05.

a,b Different superscripts in a row indicate significant difference at p<0.05.

x,y Different superscripts in a column indicate significant difference at p<0.05.

**Table 4:** Effect of the length of fasting on indexes on carcass contamination of broilers

Indexes	Raising pattern	Length of fasting (h)1)					Effect2)		
	
		0	4	6	8	10	Pattern	Length	Pattern×length
Contamination propensity (g)	Floor-feed	2.00^a^±0.14	1.60^ab^±0.13	1.00^b^±0.07	0.80^bc^±0.15	0.40^c^±0.08	-	*	-
	Scatter-feed	2.20^a^±0.18	1.80^ab^±0.29	0.80^b^±0.15	0.80^b^±0.12	0.20^c^±0.06			
Proventriculus contents (g)	Floor-feed	0.95±0.11	0.76±0.03	0.82±0.03	0.92±0.18	1.04±0.11	-	-	-
	Scatter-feed	0.72±0.10	0.79±0.02	0.85±0.06	0.74±0.02	1.13±0.16			
Gizzard contents (g)	Floor-feed	10.98^a^^y^±1.22	10.92^a^±0.63	6.87^ab^±0.97	6.86^ab^^y^±0.72	3.50^b^^y^±0.68	*	*	-
	Scatter-feed	16.89^a^^x^±0.80	10.65^ab^±0.23	8.92^b^±1.01	13.51^ab^^x^±2.29	7.39^b^^x^±0.50			
Whole intestine contents (g)	Floor-feed	32.25^a^±0.59	18.04^b^±0.39	15.12^bc^±1.28	13.66^bc^±0.49	7.61^c^^y^±0.51	*	*	-
	Scatter-feed	29.81^a^±1.07	23.28^ab^±0.46	22.60^ab^±0.64	16.26^b^±0.22	16.83^b^^x^±0.79			

1) The data are presented as means±standard error of the mean.

2) - means p>0.05, * means p<0.05.

a–c Different superscripts in a row indicate significant difference at p<0.05.

x,y Different superscripts in a column indicate significant difference at p<0.05.
